# Editorial: Advances in Facet Theory Research: Developments in Theory and Application and Competing Approaches

**DOI:** 10.3389/fpsyg.2019.01642

**Published:** 2019-07-18

**Authors:** Paul M. W. Hackett, Yael Fisher

**Affiliations:** ^1^Psychology Department, University of Gloucestershire, Cheltenham, United Kingdom; ^2^Department of Philosophy, Durham University, Durham, United Kingdom; ^3^Achva Academic College, Arugot, Israel

**Keywords:** facet theory, mapping sentence, qualitative facet theory, declarative mapping sentence, multidimensional statistics

When conducting research into human behavior we often discover that multiple independent variables impact several dependent variables. To put this is another way, in complex behavioral research the variables of interest often form an intricate interacting web. In this situation, the researcher has to adopt a research approach that embraces and considers this multiplicity of effects and outcomes: Facet Theory is one such approach. This collection represents a significant updating of Facet Theory scholarship, in which the editors have collected a series of Facet Theory articles along with a chapter that considers multidimensional data visualizations not from a facet perspective. The papers address a wide range of domains of human experience: education, homicide, work life, understanding art, and bird behavior. Multivariate research design procedures and the interpretation of multivariable research findings characterize the papers in the collection.

Facet theory is an approach to theory construction used in the social sciences, which integrates research design with data analysis (Guttman, 1959; Tziner, 1987; Borg and Shye, 1995; Hackett, 2014). A central concept in Facet Theory is the facet, which is a classification of an issue under investigation. Cardinal within Facet Theory is the Mapping Sentence, which is a tool for conceptualizing theoretical structures, planning research designs, selecting the variables for use in a study, and formulating the hypotheses to be investigated.

The mapping sentence is a tool that exists within Facet Theory that allows the design and interpretation of the output from multivariate statistical plots. The mapping sentence is an approach that is both unique to Facet Theory and unique in that it is employed from design to research interpretation stages. In all the Facet Theory articles in this Research Topic, the authors present mapping sentences for their specific research domains. In most of the articles, the mapping sentences that have been used to design the research are analyzed psychometrically using smallest space analysis (SSA), a multi-dimensional non-metric data visualization technique. In her paper, Fisher used a mapping sentence approach coupled with SSA to analyze motives for joining a parent-teacher association and compared her results with factor analysis (FA) that have previously been undertaken on the measuring instrument^1^. Fisher's SSA yielded a polarized facet of self-serving altruistic ideological motives, self-serving altruistic pedagogical motives, and egoistic motive, along with a radial form facet with elements of concealed motives and politically correct-driven motives in a Radex configuration.

Moreover, Shkoler et al. noted that several definitions and measures of workaholism existed and investigated this also using SSA and FA. They developed a questionnaire based upon a two-facet definition of workaholism and proposed a definitional model incorporating a modality facet with cognitive, emotional, and instrumental elements and a facet of resources that had two elements (time and effort). Also, in a work-related context, Tziner and Levy studied the personality, attitudes, beliefs, etc., of managers and military officers toward performance appraisal. They examined connections between raters' appraisal ratings along with measures of performance and discrimination measures. They designed a mapping sentence and their findings confirmed the presence of a polarizing facet with elements of rater, ratee, and organization/system. The structure led to them concluding that this indicated these three considerations may have been “equally proximal in determining rater behavior.”

A similar domain (workplace and personality) is the subject of de Souza and Roazzi's chapter. However, they root their understanding of personality in a fictitious model developed in sci-fi writing by Veronica Roth. In her dystopian and post-apocalyptic society, she posits five psychological traits. The authors claim that the fictional typology may provide an important contribution to personality studies. They investigate the typology's utility for understanding work life choices and within organizations' experiences. SSA supported the five-part division of personality. They concluded that the five traits were psychologically valid and predicted work life choices and experiences. In a second paper by de Souza and Roazzi, the authors (with Monica Souza) looked at possible psycho-cultural underpinnings for some murders. They investigated the Culture of Honor and how this supports ideas that men should never show weakness and how this may be associated with homicide. SSA was used to analyze Brazilian men's attitudes and discovered aspects of culture were linked to traditional masculinity, anger, and other negative personality traits which increased a propensity toward committing homicide. They claim that their approach supported a new scientific framework that clarifies psycho-cultural processes and mechanisms.

There are three chapters that present Facet Theory as a qualitative research approach. Capobianco philosophically reflects upon the book by Paul Hackett that presents the possibilities of using the mapping sentence in a qualitative and philosophically manner. Capobianco concludes that there are difficulties associated with the extra time and effort that a researcher must expend when using a Facet Theory approach. However, he concludes that these efforts are worthwhile, and that Facet Theory is valuable in both qualitative and quantitative research. The first of Hackett's two papers takes a philosophical Facet Theory perspective and uses a mapping sentence to explore and talk about abstract fine art practice. He employs characteristics of artwork suggested by Crowther's and reduces these using a Facet Theory approach and presents these in a mapping sentence with facets of resemblances, novel environments, visual suggestions, and spatiality/structure. In his second article Hackett considers both quantitative and qualitative facet approaches and applies these to investigate birds' cognitive performance in the wild. Through SSA and partial order scalogram analysis (POSA) he claims Facet Theory can be used to design research into avian cognition research and that the structure of avian intelligence may be understood using a mapping sentence.

Payton Jones and colleagues take a non-Facet Theory approach to multivariate research. Their paper claims multidimensional data visualizations may be misunderstood. In Jones et al., researchers consider their aims when they evaluate the strengths and weaknesses of different visualization approaches. They note how whilst many methods exist that produce interpretable visualizations of highly complex data in two dimensions, two-dimensional representations of high-dimensional data are never able to fully depict its complexity. They suggest researchers should give the reasons for their choice of approach and also provide details as to how their particular visualizations can be understood. In another non-Facet Theory article, Segalowitz et al. investigate the use of multidimensional scaling approaches (MDS) in neoteric combinations with other techniques. They focused upon looking at the comprehension of language that expresses doubt and uncertainty in clinically located health communications. MDS approaches used factor analysis, Classical-MDS, and cluster analysis and discovered a three-dimensional semantic space that produced interpretable results in relation to language usage. They concluded their results supported the use of MDS to assess word understanding in the context of health communication.

This Research Topic provides a review of contemporary Facet Theory and multidimensional data visualization in quantitative, qualitative, and philosophical research. We thank the contributors and hope that this open, online Research Topic will enable the younger generation of researches to better understand this unique theory and expand the possibilities of research methodologies available to them.

## Author Contributions

All authors listed have made a substantial, direct and intellectual contribution to the work, and approved it for publication.

### Conflict of Interest Statement

The authors declare that the research was conducted in the absence of any commercial or financial relationships that could be construed as a potential conflict of interest.

## Footnotes

^1^The facet approach is often used to analyze an existing domain.
